# Effects of High-Fat Diet Induced Obesity and Fructooligosaccharide Supplementation on Cardiac Protein Expression

**DOI:** 10.3390/nu12113404

**Published:** 2020-11-05

**Authors:** Sidra Sarfaraz, Shamjeet Singh, Aileen Hawke, Sandra T. Clarke, D. Dan Ramdath

**Affiliations:** 1 Guelph Research and Development Centre, Agriculture and Agri-Food Canada, Guelph, ON N1G 5C9, Canada; sidra9518@gmail.com (S.S.); aileen.hawke@canada.ca (A.H.); 2 School of Pharmacy, Faculty of Medical Sciences, The University of the West Indies, St. Augustine, Trinidad and Tobago; shamjeet.singh@sta.uwi.edu; 3 Applied Bioscience Graduate Program and Faculty of Science, University of Ontario Institute of Technology, Oshawa, ON L1G 0C5, Canada; sandra.clarke2@canada.ca

**Keywords:** cardiac, proteomics, mass spectrometry, DAVID, KEGG, fructooligosaccharides, prebiotic, CVD

## Abstract

The mechanism by which high fat-diet induced obesity affects cardiac protein expression is unclear, and the extent to which this is modulated by prebiotic treatment is not known. These outcomes were assessed in rats initially fed a high-fat diet, then the top 40% weight gain group were randomly allocated to control (CON), high-fat (HF) and HF supplemented with fructooligosaccharide (32 g; HF-FOS) treatments for 12 weeks (*n* = 10/group). At sacrifice, left ventricles were either frozen or preserved in formalin. Serum was stored for glucose and insulin measurements. Protein spectra was obtained using an Orbitrap analyzer, processed with Sequest and fold changes assessed with Scaffold Q +. Treatment effects for body weights, glucose and insulin were assessed using one-way ANOVA, and the differential protein expression was assessed by a Mann–Whitney U test. The Database for Annotation, Visualization and Integrated Discovery and the Kyoto Encyclopedia of Genes and Genomes identified pathways containing overrepresented proteins. Hematoxylin and eosin sections were graded for hypertrophy and also quantified; differences were identified using Chi-square analyses and Mann-Whitney U tests. HF diet fed rats were significantly (*p* < 0.05) heavier than CON, and 23 proteins involved in mitochondrial function and lipid metabolism were differentially expressed between HF and CON. Between HF-FOS and HF, 117 proteins involved in contractility, lipid and carbohydrate metabolism were differentially expressed. HF cardiomyocytes were significantly (*p* < 0.05) more hypertrophic than CON. We conclude that high-fat feeding and FOS are associated with subcellular deviations in cardiac metabolism and contractility, which may influence myocardial function and alter the risk of cardiovascular disease.

## 1. Introduction

Cardiovascular diseases (CVDs) continue to be the leading cause of morbidity and mortality globally [1]. While some genetic markers have been identified, they explain less than 15% of the variance in the risk for these diseases [2]. It is well accepted that external factors, such as poor diets, sedentary lifestyles and obesity are the major contributors to CVD risk [3,4,5]. In particular, obesity contributes significantly to diminished cardiovascular function and increased CVD risk [6]. Cardiac dysfunction associated with obesity is often accompanied by systemic abnormalities such as chronic inflammation, oxidative stress, dyslipidemia, insulin resistance and endothelial dysfunction [7]. On a high fat diet, obese animals display cardiomyocyte hypertrophy, myocardial interstitial fibrosis [8] and disrupted signal transduction in cardiomyocytes, which decrease the ability of these cells to efficiently utilize glucose and lipids [9]. Furthermore, early stage cardiomyopathy caused by high-fat induced obesity in mice is associated with disturbed calcium homeostasis [5]. In humans, increased intake of saturated fatty acids has been shown to promote a pro-inflammatory state and is associated with insulin resistance in muscle and fat tissues [10].

Collectively, biochemical abnormalities associated with high fat diets can lead to cardiac contractile impairments and adaptive myocardial remodelling, which in turn may result in diminished cardiac contractility and an increased risk of heart failure [4,11]. When a proteomic analytical approach was used, insulin resistant mice fed a high fat diet had decreased expression in proteins involved in heart structure, energy metabolism, fat oxidation and the tricarboxylic acid cycle (TCA) cycle [12]. These findings confirm a direct effect of high fat diet on cardiac function, which can be implicated in an increased cardiovascular disease risk. There is now an emerging field of research that suggests the gut microbiome may modulate the relationship between consumption of a high fat diet and the onset of obesity and cardiovascular disease [13]. It is likely that this modulatory effect is mediated through changes in circulating metabolites, but it is not clear what effect this has on tissue level protein expression.

The human gut microbiota is composed of more than 10^14^ bacteria and archaea as well as viruses, fungi, and protozoa. Its variability is affected by genetics, lifestyle and especially dietary components such as prebiotics and probiotics [14]. Prebiotics, or non-digestible food components such as inulin and, fructooligosaccharides (FOS) are associated with favourable modulation of the gut microbiota, and decreased post-prandial glucose and insulin [15,16]. A recent meta-analysis demonstrated that prebiotic intakes are associated with decreases in body mass index (BMI), body weight and fat mass in humans [17]. Review of current evidence also suggests that consumption of prebiotics is inversely associated with obesity, which may be mediated through decreased inflammation and increased production of short chain fatty acids (SCFAs) [18]. It is possible that changes in concentrations of these metabolites may modulate the expression of proteins, and in the case of cardiac function may be associated with a lowering of CVD risk.

This study was undertaken to investigate the effect of high fat-diet induced obesity on cardiac protein expression using a proteomic approach, and to assess the protective effect of supplemental FOS. We hypothesized that obesity would result in impairments in cardiac proteins involved in contractility, insulin signaling and cellular metabolism, and that FOS supplementation would augment these impairments.

## 2. Materials and Methods

### 2.1. Experimental Design and Sample Collection

Weanling male Sprague Dawley rats approximately 28 days old were obtained from Charles River (Saint Constant, QC, Canada). Rats were pair-housed in Sealsafe^®^ PLUS Rat individually ventilated cages (IVC, Techniplast, Via I Maggio, Italy) equipped with wire mesh bottoms, gnawing sticks, covered tunnels, and stainless-steel sheets for environmental enrichment, and health checks were performed daily. Room temperature was maintained at 22 °C with 12-hour light/dark cycles, and rats were provided chow and water *ad libitum*.

After an acclimation period of seven days, all rats were initially placed on a high-fat modified AIN-93G diet for two weeks to identify rats that were resistant or susceptible to an obesogenic diet. Rats in the top 40% weight-gain category were categorized as the diet-induced obesity (DIO) phenotype. Following two weeks of high-fat diet consumption and distinction of the obese phenotype, DIO rats (*n* = 10 per group) were further separated into new diet categories, either receiving the base AIN-93G chow as a control diet (CON), a high-fat modified AIN-93G diet (HF), or a high-fat modified AIN-93G diet containing 32.2 g of fructooligosaccharide/1 kg diet (HF-FOS). The amount of FOS included in the test diet (3%) can be reasonably achieved within the human population [19]. The control diet contained 70 g total fat/kg diet and the HF-modified diets each contained 165 g total fat/kg diet. Detailed composition of the different diets is shown in Table 1.

Following 12 weeks of experimental diet feeding, necropsies were conducted. Rats were humanely euthanized under isoflurane anesthesia by terminal cardiac bleeds. Non-fasted blood samples were distributed in BD Vacutainer^®^ SST™ Tubes (BD, Franklin Lakes, NJ, USA) and processed according to the manufacturer’s directions. Serum was used immediately for glucose measurements or stored at −80 °C for future analyses. Fasted samples (beyond 3 hours) were not possible for this experiment as it would have impacted other critical measures of the major study. Following blood draws, the heart was excised, and the left ventricular tissue was collected, washed in cold PBS and a portion was immediately cryopreserved in liquid nitrogen for protein analysis by mass spectrometry at the SPARC BioCentre (SickKids Hospital, Toronto, ON, Canada). The other portion of the heart was fixed in 10% neutral-buffered formalin at ambient temperature for histological analysis at the Department of Preclinical Sciences, the University of the West Indies, Trinidad. All animal care procedures were approved by the animal care committees at Health Canada and at the University of Ontario Institute of Technology.

### 2.2. Serum Insulin and Glucose Measurements

Serum glucose concentrations were measured on the day of necropsy using a glucose test kit (Ortho-Clinical Diagnostics, Markham, ON, Canada) on a VITROS^®^ 5,1 FS automated clinical chemistry analyser. Circulating insulin concentrations were determined from previously frozen serum samples using a Rat Metabolic Bead Panel Milliplex Map Kit (EMD Millipore Corp. Billerica MA, USA) on a Luminex 200 analyser (Luminex, Austin, TX, USA). There were two levels of commercial quality controls on each Luminex assay plate, and insulin concentrations were found to be within the expected range.

### 2.3. Histology

Formalin preserved tissue was oriented and embedded in paraffin wax. Sections (7 µm) were cut cross-sectionally and stained with haematoxylin and eosin (Sigma-Aldrich, MO, USA) to visualise tissue morphology. Micrographs were taken at a magnification of 40 × and assessed qualitatively for hypertrophy (cardiomyocyte size) and evidence of interstitial (collagen) deposition/extracellular matrix depositions using four categories: no-evidence, mild, moderate or severe. Chi-squared analyses were used to detect significant associations between cardiac hypertrophy and interstitial deposition and the treatment groups. Micrographs were also quantified using ImageJ2. Briefly, images were converted to 16 bit, and thresholds were used to estimate the percentage of the micrograph occupied by the cardiomyocyte. Mann–Whitney U tests were used to detect any significant differences in cardiomyocyte area between CON and HF and HF and HF-FOS. A *p*-value of < 0.05 was considered significant.

### 2.4. Mass Spectrometry

Mass spectrometry analysis was performed at the SPARC BioCentre at the Hospital for Sick Children in Toronto, ON Canada. Cryopreserved cardiac tissue was sliced, incubated with 1 mL cold RIPA (1% Triton X-100, 1% sodium deoxycholate, 0.1% sodium dodecyl sulfate, 150 mM NaCl, 50 mM Tris pH = 7.5, 1 mM EDTA buffer) and homogenised using a TissueLyser LT (Qiagen, Inc., MD, USA). The homogenate was centrifuged at 23,000 × *g* for 15 minutes, and protein concentrations estimated (Pierce™ BCA Protein Assay Kit, ThermoFisher Scientific, MD, USA) using bovine serum albumin as a standard. Homogenate (100 μg) was mixed with 100 mM triethylammonium bicarbonate (TEAB) to make a final volume of 100 μL. Each protein digest (50 µg) was then labelled with TMT tags and placed into one of three groups of 10; groups were divided to include at least three random samples from each dietary group (CON, HF, HF-FOS) per 10-plex. Samples were cleaned using Strong Cation Exchange clean up tips. Peptides were bound to strong cation exchange material, washed, and eluted off in two stages/TMT group. Each elution was then lyophilised and individually analyzed by LC-MS/MS.

Samples were analyzed on an Orbitrap mass spectrometer (Q-Exactive, ThermoFisher, San Jose, CA, USA) using the method outlined by Ayoub et al. with some modifications [20]. Peptides were eluted over 180 min at a rate of 250 nL/min using a gradient set up as 0%–40% of buffer A (0.1% formic acid) and buffer B (0.1% formic acid in 80% acetonitrile). The method included one MS full scan (525–1600 m/z) followed by 15 data-dependent MS/MS scans with a resolution of 35,000. Fragmentation occurred in the higher energy collisional dissociation (HCD) trap with normalized collision energy set to 27V.

### 2.5. Protein Identification and Quantitation

All MS/MS samples were analyzed using Sequest (Thermo Fisher Scientific, San Jose, CA, USA; version 1.4.1.14) and X! Tandem (version CYCLONE (2010.12.01.1)). Peptide probabilities from Sequest were assigned by the Scaffold Local FDR (false discovery rate) algorithm [21]. Peptide Probabilities from X! Tandem were assigned by the Peptide Prophet algorithm [22] with Scaffold delta-mass correction. Scaffold Q + (Proteome Software Inc., Portland, OR; version Scaffold_4.7.3) was used to quantitate peptide and protein identifications. Protein identification was accepted if it could be established at greater than 95.0% probability and contained at least two identified peptides. Normalisation was performed as described previously [23].

### 2.6. Statistical Analyses

This work represents secondary analyses of tissues provided from a larger animal study and characterizes heart muscle collected from a subset of 10 obese rats per diet group. Analyses sought to detect significant differences between (i) HF vs. CON and (ii) HF-FOS vs. HF experimental groups. Glucose, insulin and body weights were normally distributed and analyzed by one-way ANOVA followed by a post-hoc Tukey test. For insulin measures only, there was a sample number of 5 due to constraints on analyzing serum metabolic hormones for the larger study. Fold changes between these groups were calculated by Scaffold Q +. Differentially expressed proteins were determined by using Mann–Whitney U tests, with unadjusted significance level *p* < 0.05 corrected by Benjamini–Hochberg.

### 2.7. In-Silico Functional Analyses

In silico methods were used to establish the possible functional relationships between differentially expressed proteins in HF vs. CON hearts and HF-FOS vs. HF hearts. The official gene names were utilised in the Database for Annotation, Visualization and Integrated Discovery (DAVID) [24] to perform functional enrichment analyses and elucidate the biological processes where differentially expressed proteins were overrepresented. The enriched Kyoto Encyclopedia of Genes and Genomes (KEGG) pathways and gene ontology (GO) terms for biological processes in the list of the differentially expressed proteins were identified. The *p*-values of these enrichment analyses were adjusted using Benjamini–Hochberg (*p* < 0.05).

## 3. Results and Discussions

### 3.1. Obesity

**Figure 1:**
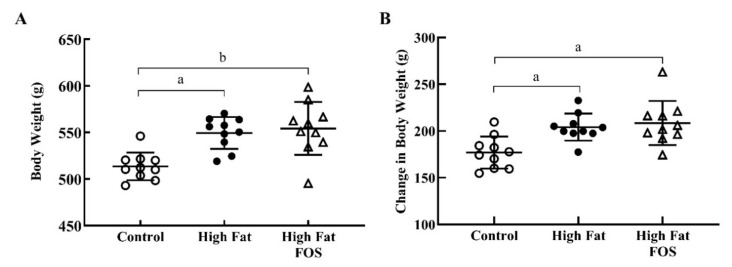
Body weights (mean ± standard deviation) for control, high-fat, and high fat- fructooligosaccharide (FOS) (*n* = 10 each group). (**A**) Total body weight after 12 weeks on experimental diets. (**B**) Change in total body weight after 12 weeks on experimental diets. (a) Tukey-adjusted *p*-value < 0.01; (b) Tukey-adjusted *p*-value < 0.001.

At the end of this 12-week study, HF diet-fed rats were significantly heavier (*p* < 0.05; 549.4 ± 17.1 g) than CON rats (513.6 ± 14.8 g; Figure 1A), and FOS supplementation (554.3 ± 28.4 g) did not attenuate the obesity phenotype. HF diet-fed rats with or without FOS supplementation gained more weight during this period than CON rats (*p* < 0.01; Figure 1B). This finding is in contrast to results of a recent meta-analysis of human trials that concluded prebiotic consumption was associated with significant decreases in BMI and body weight. It has been suggested that products of FOS fermentation (e.g., SCFA) can modulate glucagon-like peptide-1 (GLP-1) secretion [25], and therefore, influence satiety and weight gain [26]. However, it is possible that the dose of FOS used in this study may not have been sufficiently high; anti-obesogenic effects of FOS have been demonstrated at 5% to 20% [18]. The dose used in our current study was not based on the efficacy of FOS to prevent obesity; rather it is based on a previous human study that examined changes in gut microbiota [19].

### 3.2. Glucose and Insulin

**Figure 2:**
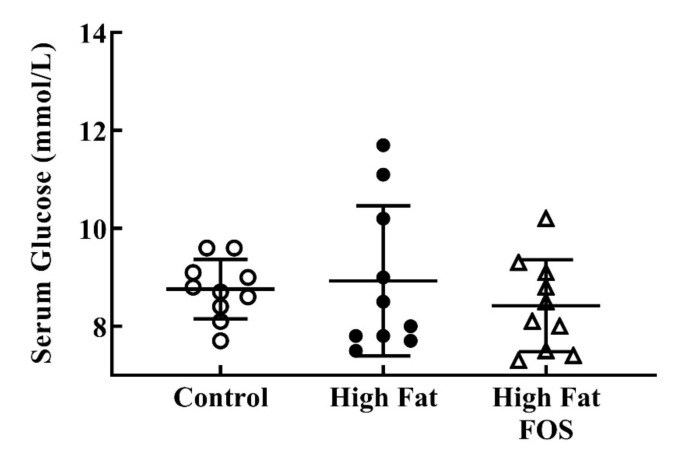
Non-fasting serum glucose (mmol/L) (mean ± standard deviation) for control, high-fat, and high fat-FOS (*n* = 10 each group) after 12 weeks were not significant by ANOVA.

**Figure 3:**
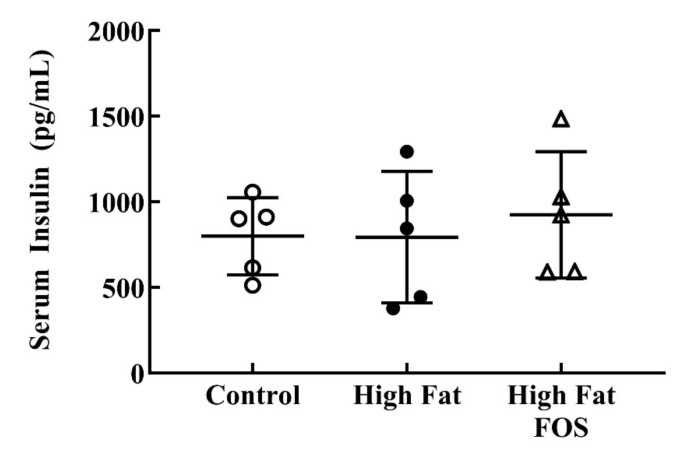
Non-fasting serum insulin (mean ± standard deviation) for control, high-fat, and high fat-FOS (*n* = 5 each group) after 12 weeks were not significant by ANOVA. Glucose and insulin were not measured for all animals in a group.

Serum glucose (Figure 2) and insulin (Figure 3) concentrations between experimental groups were not significantly different. This may be attributed to the blood samples not being obtained in the fasting state. Additionally, the dose of FOS used in the diet may have been too low to produce significant changes in these measures.

### 3.3. Histology

**Figure 4:**
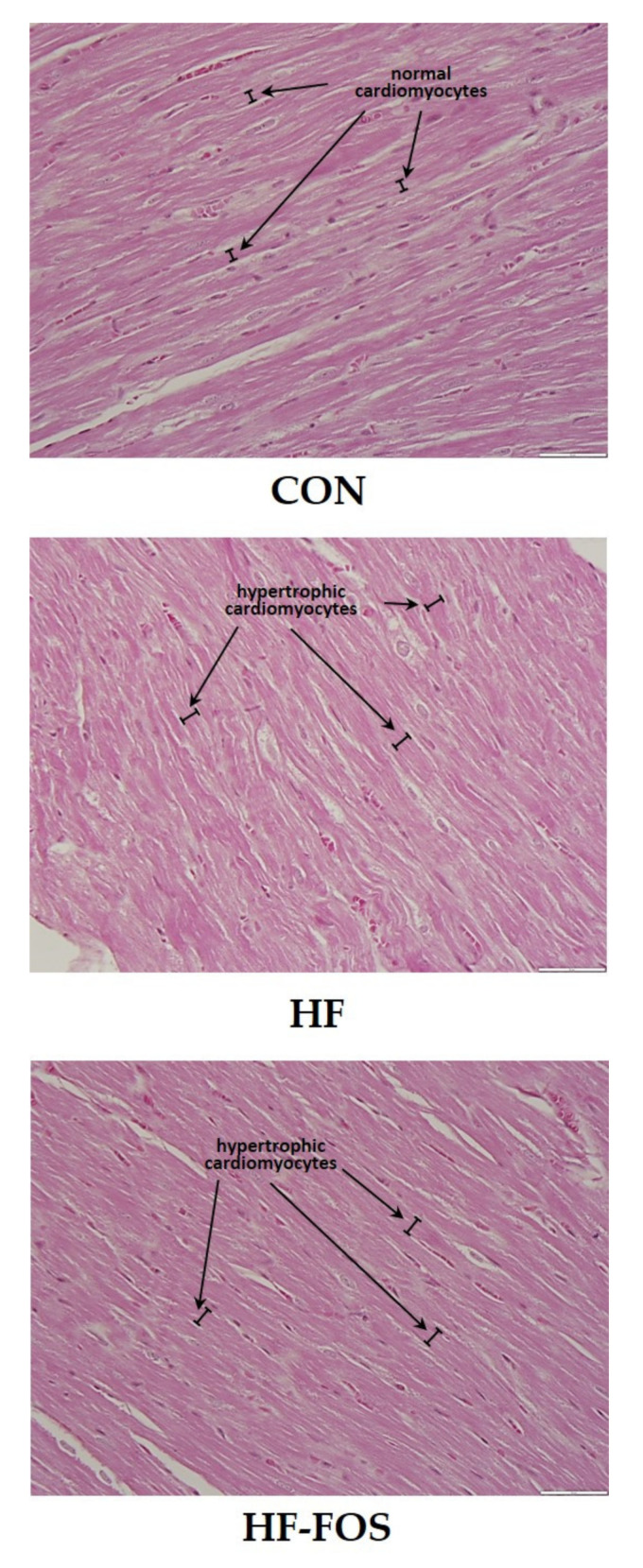
Sample micrographs (40 ×) of left ventricular tissue from CON, HF and HF-FOS groups with representative cardiomyocytes identified. CON, control diet; HF, high-fat diet; HF-FOS, high-fat diet supplemented with fructooligosacchride.

**Figure 5:**
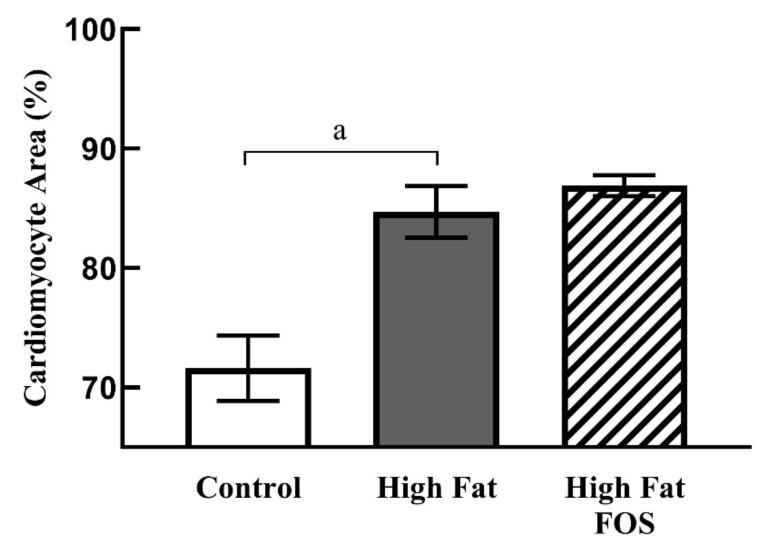
Cardiomyocyte area (mean ± SD) as a percentage of total area from left ventricular tissue in CON, HF and HF-FOS groups. Quantitation was done using ImageJ2. CON, control diet; HF, high-fat diet; HF-FOS, high-fat diet supplemented with fructooligosacchride; **a**: significantly different at *p* < 0.05; *n* = 7.

Figure 4 shows representative micrographs, with representative cardiomyocytes identified in cardiac tissue from rats on CON, HF and HF-FOS diets after 12 weeks of feeding. As shown in Figure 5, qualitative assessment revealed that HF diet was associated with hypertrophic cardiomyocytes (*p* < 0.001), but not increased interstitial fibrotic depositions, and hypertrophy was not attenuated by prebiotic treatment. This qualitative assessment was confirmed by quantification of cardiomyocyte area using Image J2 (*p* < 0.05). Cardiomyocyte area (mean percentage ± SD) was significantly increased (*p* < 0.05) in HF heart (84.69 ± 2.15) when compared to CON hearts (71.60 ± 2.75); this was not reversed by prebiotic treatment. Cardiomyocyte hypertrophy is a noted consequence of obesity [27], but increased cardiac fibrosis following HF has mainly been reported in long-term studies ranging over 7–16 months [28,29]. Although not measured directly in this study, it is likely that cardiac dysfunction would have resulted from HF feeding over a longer period of time [4].

### 3.4. Cardiac Tissue Protein Expression

Of the 52,664 spectra obtained via mass spectrometry, 45,730 (87%) were quantitated at the thresholds used for this study, and 1501 proteins were identified and quantified (Supplemental Table S1). Compared to CON, HF treatment resulted in significant changes in protein expression: 10 were upregulated and 13 were downregulated (Supplemental Table S2). Five KEGG pathways and 11 GO biological processes were identified by DAVID (Table 2). Between the HF and HF-FOS groups, 117 proteins were significantly different with 96 downregulated and 21 upregulated (Table 3). DAVID identified 6 different GO biological processes that were significantly enriched (Table 4).

#### 3.4.1. Lipid Metabolism (HF vs. CON Diet): Supplemental Table S2 and Table 2

The expression of several proteins involved in lipid metabolism were affected by HF treatment apolipoprotein A-IV (APOA4) and preproapolipoprotein A-I (apolipoprotein A-I; APOA1) proteins were upregulated. In both animal and human experiments, increased APOA4 was associated with decreased atherogenicity [30,31] and increased apolipoprotein B to APOA1 ratio correlated with congestive heart failure in overweight and obese patients [32]. Therefore, an upregulation of these apolipoproteins may reflect a protective response following HF treatment.

Acyl-CoA synthetase family member 2, mitochondrial precursor (ACSF2) is involved in fatty acid synthesis [33] and was downregulated following HF treatment. In contrast, enoyl coenzyme A hydratase 1 (peroxisomal) (ECH1), which is involved in the beta-oxidation of fatty acids, was upregulated on HF presumably due to increased substrate flux. An upregulation of this enzyme has been shown to be protective in rodent models of obesity [34]. In addition to the aforementioned changes, the upregulation of hydroxysteroid (17-beta) dehydrogenase 4, isoform CRA b, partial (HSD17B4) (Supplemental Table S2 and Table 2), which is involved in the formation of 3-ketoacyl-CoA intermediates, suggests that more acetyl-CoA units were produced by fatty acid catabolism following HF treatment, but not utilised for ATP production.

In cardiac tissue, increased beta-oxidation has been suggested to be both adaptive and pathological. This is because lipid uptake can become toxic for the heart when excess fatty acids are converted to diacylglycerols or ceramides, both of which have roles in cardiac dysfunction and insulin resistance [35]. We have shown that HF feeding by obese rats over 12 weeks results in decreased expression of proteins associated with fatty acid synthesis and an increase in those involved in beta-oxidation. Pellieux et al. demonstrated that healthy mice had increased fatty acid oxidation proteins after HF feeding [36]. However, whether HF induced cardiomyopathy occurs because of increased fatty acid oxidation or lipid accumulation remains to be clarified [37].

Several proteins involved in ubiquinone metabolism were decreased by HF feeding; these included coenzyme Q9 partial (COQ9), ADCK3, mitochondrial isoform X1 (COQ8A) and electron transferring flavoprotein, alpha polypeptide (ETFA). Increased dietary COQ9 was shown to improve left ventricular performance, reduce myocardial infarct size and reduce cardiomyocyte apoptosis [38]. The observed reductions in these three proteins could reflect compromised cardiac function via disruption of oxidative phosphorylation, potentially leading to a reduction in ATP synthesis. Similarly, creatine kinase, brain isoform CRA_b (CKB), which catalyzes the transfer of phosphate between ATP and creatine, was downregulated (Table 2). This may be a protective adaptation since excessive myocardial creatine is associated with cardiac hypertrophy, dilatation, impaired contractile function [39] and impaired energy generating pathways including reduced glycolytic function [40].

#### 3.4.2. Protein Metabolism (HF vs. CON Diet): Supplemental Table S2 and Table 2

Isovaleryl coenzyme A dehydrogenase, isoform CRA_a (IVD), RCG20683, isoform CRA_b, (maleylacetoacetate isomerase) (GSTZ1), Aldh4a1 protein, partial (ALDH4A1) and adenylosuccinate synthetase isozyme 1 (ADSSL1) proteins were all downregulated in the HF diet group (Table 2 and Supplemental Table S2). These proteins are all involved in various aspects of amino acid metabolism [41] with the exception of *Adssl1*, which encodes for a muscle-specific enzyme that catalyzes the first step in the conversion of IMP to AMP. As such, decreased production of these proteins suggest that HF treatment might have diminished amino acid catabolism and compromised energy production, likely in favour of fatty acid catabolism.

#### 3.4.3. Oxidative Stress (HF vs. CON Diet): Supplemental Table S2 and Table 2

In terms of the regulation of ROS, catalase isoform CRA_b (CAT) was upregulated while tripartite motif protein 50 (predicted) (TRIM72) was downregulated in the HF group. The change in CAT expression is consistent with other studies that employed HF feeding. Increased fatty acid oxidation leads to more mitochondrial H_2_O_2_ production, and subsequently larger amounts of catalase is needed [42]. The decreased production of TRIM72 is contrary to this finding, since it is a membrane repair protein that is activated by the type of membrane damage that occurs during ischemic reperfusion injury or with exposure to oxidants such as H_2_O_2_ [43]. In hearts of a transgenic type I diabetes mellitus model, increased expression of CAT preserved normal cardiac morphology, prevented contractile defects, and reduced malondialdehyde-modified proteins. These authors also demonstrated that the source of the ROS and oxidative damage was the functioning mitochondria [44]. The NAD(P) transhydrogenase, mitochondrial isoform X1 (NNNT) protein regenerates NADPH that is utilised by glutathione peroxidase (GPX) and the thioredoxin/peroxiredoxin systems to convert H_2_O_2_ to H_2_O and optimise ROS balance during cellular respiration [45]. Its downregulation in this experiment matches the trend seen with TRIM72, where ROS regulatory proteins were downregulated.

#### 3.4.4. Cellular Growth and Proliferation (HF vs. CON Diet): Supplemental Table S2 and Table 2

Expression of several proteins involved in cellular growth and proliferation was affected by HF treatment: Annexin A6 isoform X1 (ANXA6), a calcium dependent protein involved in cardiac contractility, was decreased, while there were increases in Filamin, alpha (predicted), isoform CRA_b (FLNA), RCG49564, isoform CRA_a (Cadherin-2) (CDH2) and Stress-70 protein, (heat shock protein-70) mitochondrial (HSPA9). These all have different roles in cell membrane structure, proliferation and aging [46]. FLNA links actin to membrane glycoprotein and is therefore involved in cytoskeletal effects such as changes in cell shape, cell migration and adhesion. Altered expression of *AnxA6* might impair cardiac contraction and relaxation cycles [47]. CDH2 comes from a superfamily of adhesion molecules that mediate Ca^2+^- dependent cell-to-cell adhesion in tissues [48]. In a model of myocardial hypertrophy, CDH2 was postulated as one of the mechanisms that enables the heart to maintain its physical structure and mechanical function by enhancing cell-to-cell contact and membrane connections [49]. Mitochondrial HSPA9 is a chaperone protein in the biogenesis and refolding of mitochondrial iron-sulfur proteins; it may also have a role in cell proliferation and cellular aging [46]. While the biochemistry of myocardial remodeling remains a mystery, these changes may reflect initial adaptations in cell growth, proliferation and migration consistent with the hypertrophy observed in hematoxylin and eosin stained tissues, and may eventually threaten cardiac compliance.

#### 3.4.5. Lipid Metabolism (HF-FOS vs. HF Diet): Table 3 and Table 4

Several proteins involved in the fatty acid homeostasis cluster were upregulated in the HF-FOS group compared to the HF group, with the exception of isobutyryl-CoA dehydrogenase (ACAD8) and apolipoprotein A4 (APOA4). HF rats had greater APOA4 levels compared to CON whereas FOS supplementation downregulated APOA4, which may have resulted in decreased intestinal lipid absorption [50] and/or altered fatty acid oxidation. Further, it was shown that SCFA infusion results in reduced APOA4 synthesis compared to long chain fatty acids [51]. Therefore, increased bacterial SCFA production following FOS supplementation may have downregulated APOA4, but it is unclear what impact this would have on cardiac function. HF-FOS also altered several acyl-coA dehydrogenases. While ACAD8 was downregulated, the upregulation of other dehydrogenases such as acyl-coenzyme A dehydrogenase, very long chain, isoform CRA_c (ACADVL) and acyl-CoA dehydrogenase short chain (ACADS) in the HF-FOS group indicates increased rates of beta-oxidation [52]. The upregulation of acyl-coA dehydrogenases, which require electron transfer flavoprotein alpha subunit (ETFA) as an electron acceptor [53], was associated with increased ETFA observed in HF-FOS hearts. Collectively, these changes point to increased oxidation of fatty acids, presumably as a result of high substrate availability from HF diets, and this was potentiated by FOS supplementation and the action of the gut microbiota.

Fermentation of FOS by the gut microbiota, specifically bifidobacteria [54], produces acetate and lactate which can then be interconverted to butyrate [55]. Butyrate that is leftover from colonocyte metabolism is transported to the liver and peripheral tissues, where it reduces lipid synthesis and increases beta-oxidation [56]. This is similar to the results seen with HF feeding in our study; however, FOS supplementation is expected to have an additive effect via the production of SCFAs, which would likely further reduce the synthesis of diacylglycerols or ceramides and protect against cardiac dysfunction and insulin resistance [35].

#### 3.4.6. Protein Folding (HF-FOS vs. HF Diet): Table 3 and Table 4

FOS-treatment downregulated all proteins in the protein folding cluster. Specifically, FOS supplementation decreased the expression of antioxidant and chaperone-like proteins, which tend to increase with obesity or diabetes. This reduction may be due to lower cellular stress in FOS-treated animals [57,58].

#### 3.4.7. Adhesion and Cytoskeletal Regulation (HF-FOS vs. HF Diet): Table 3 and Table 4

Most adhesion and cytoskeletal proteins also decreased with FOS. Specifically, eukaryotic translation elongation factor 1 delta (EEF1D) and drebrin-like protein (DBNL) were decreased with FOS treatment, whereas spectrin, alpha, non-erythrocytic 1 (SPTAN1) was increased. By upregulating SPTAN1, FOS treatment may have enhanced cardiomyocyte cell adhesion, and the transmittance of force between contractile proteins and the extracellular matrix [59], thereby promoting cardiac contractility.

#### 3.4.8. Cardiac Muscle Regulation (HF-FOS vs. HF Diet): Table 3 and Table 4

The HF-FOS group showed changes in several contractile proteins compared to the HF group. Calsequestrin 2 (CASQ2), which interacts with ryanodine receptors and modulate the duration of calcium efflux from the sarcoplasmic reticulum [60,61], decreased with FOS supplementation. While calcium handling and ryanodine receptor dysregulation are characteristics of cardiac pathologies, including heart failure [60], the specific role of CASQ2 in these diseases remains unclear. HF treatment alone did not result in a change in CASQ2 after 15 weeks [62], therefore the decrease found in our study is likely attributed to FOS and may protect against cardiac hypertrophy and failure [63]. However, further research into CASQ2 and its role in regulating sarcoplasmic calcium handling is needed to clarify whether the CASQ2 reduction seen in the HF-FOS group is advantageous.

Sarcomeric and cytoskeletal proteins were also altered in the HF-FOS group. Myosin binding protein C, cardiac (MYBPC3) was higher in the HF-FOS group compared to HF. MYBPC3 determines the number of myosin heads that can interact with actin [64,65] and during diastole, it decreases myosin-actin interaction to ensure complete relaxation [66]. Myosin light chain 2 (MYL2) is another protein associated with the myosin complex [67], and was upregulated in the HF-FOS group relative to HF. While FOS upregulated MYL2 levels, it is a reduction in MYL2 phosphorylation that is implicated in cardiac diseases [67]. The present study did not assess MYL2 phosphorylation, but increased levels may contribute to stability of the myosin complex. Hearts from FOS-supplemented rats also had lower expression of myosin heavy chain 7 (MYH7), also known as myosin heavy chain (MHC)-β. This protein has been shown to be elevated in diabetes due to a switch in the myosin isozyme from MHC-α to the less-efficient MHC-β [67,68]. Heightened levels of MHC-β hinder actin-myosin kinetics and increase the chance of cardiac dysfunction [67]. Therefore, the reduction in MHC-β seen in the HF-FOS group may be beneficial to cardiac function. The expression of myomesin1 and myomesin-2 isoform X1 (MYOM1 and MYOM2, respectively), found in the M-band of the sarcomere, were increased with FOS supplementation and likely improve myosin stability [69,70,71].

Expression of cysteine and glycine rich protein 3 (CSRP3) and actinin alpha 2 (ACTN2) were both changed with HF-FOS treatment. Both proteins are found in the Z-band, the protein complex that separates adjacent sarcomere units and anchors the sarcomere to the sarcolemma. A decrease in the ratio of cytoplasmic to nuclear CSRP3 correlates with impaired mechanosensing seen in heart failure [72]. Since ACTN2 is associated with thin filament stability, its upregulation could result in improved cardiac contractility [73].

#### 3.4.9. Carbohydrate Metabolism (HF-FOS vs. HF Diet): Table 3 and Table 4

FOS supplementation was associated with decreases in almost all proteins in the carbon metabolism cluster. In the mitochondria, glutamate dehydrogenase 1 (GLUD1) forms a tri-enzyme complex with glutamic-oxaloacetic transaminase 1 (GOT1) and malate dehydrogenase [74]; GOT1 and GLUD1 levels both decreased with HF-FOS feeding in the present study. IDH3A, a subunit of isocitrate dehydrogenase 3 associated with NADH production in the TCA cycle [75], was slightly decreased with FOS supplementation. Given our consistent finding of increased fatty acid oxidation associated with the HF diet, it is reasonable to expect that NADH production via the TCA cycle, and therefore GOT1, GLUD1 and IDH3A would decrease. Succinate-CoA ligase ADP-forming beta subunit (SUCLA2) also functions in the TCA cycle. FOS was shown to increase intestinal succinate production in mice fed a HF/high-sucrose diet [76], and while the authors suggested that most of the succinate was metabolized in the cecum, our data indicates that FOS may also increase succinate within the heart. This increase in succinate supply could reduce TCA-production of succinate, causing the observed downregulation in SUCLA2 and other upstream TCA enzymes. Collectively, our data suggests that FOS affects metabolism by reducing TCA enzymes during HF feeding.

Glyoxalase 1, hydroxyacyl glutathione hydrolase (GLO1 and HAGH, respectively) and glutathione form the glyoxalase system that degrades methylglyoxal. Methylglyoxal is a by-product of glycolysis that leads to apoptosis and the production of advanced glycation end products (AGE) and ROS, and increases in diabetes [77,78] along with GLO1 activity and expression [79]. The decrease in GLO1 with FOS supplementation indicates that FOS could reduce inflammation and oxidative stress, resulting in less AGE production. A clinical trial is currently underway to determine whether inulin/FOS will decrease AGE formation in pre-diabetic adults [80]. Phosphoglucomutase 1 (PGM1) and glycogen phosphorylase, muscle (PYGM) are both involved in glycogen metabolism. PYGM breaks down glycogen into glycogen-1-phosphate while PGM1 carries out the reversible conversion of glucose-1-phosphate to glucose-6-phosphate. It is difficult to ascertain whether glycogen metabolism was up or downregulated with FOS, given that HF-FOS fed rats had greater levels of PYGM, but lower levels of PGM1. PGM1 was upregulated in skeletal muscle from diabetic patients [81], and glycogen accumulation is common in diabetic cardiomyocytes [82]. However, the role of glycogen in cardiac metabolism has yet to be fully understood [82] and thus we cannot discern whether the FOS-induced changes in PGM1 and PYGM would improve cardiac function.

## 4. Conclusions

We have attempted to reconcile the numerous changes in cardiac protein expression detected in the obese state with and without dietary FOS supplementation. Cardiomyocyte hypertrophy may be one of the earliest morphological changes. HF dietary treatment consistently resulted in increased fatty acid oxidation and reduction of energy production pathways; these were potentiated by FOS consumption. It is evident that obesity alters cardiac protein expression, including those associated with contractility, and some of these changes are modulated by FOS treatment. Importantly, these changes were not associated with significant differences in non-fasting blood insulin or glucose among experimental groups. Further research may elucidate the proteins and biochemical mechanisms that mediate these changes and predispose the heart to contractile failure. While a 12-week duration is longer than average prebiotic studies [83], extending the study period may help determine whether the observed fold changes translate to functional improvements at the organ level. Furthermore, a longer study duration may be more representative of the chronic nature of obesity and thus provide a clearer understanding of whether FOS mediates obesity-induced cardiac changes. Future research could also evaluate post-translational modifications, such as phosphorylation, which may be more indicative of cardiac function compared to protein abundance. Additionally, an assessment of the microbiome could support the proteomic findings, though studies have found an increase in *Bifidobacterium* with FOS supplementation in healthy adults [83]. To our knowledge, this is one of the first studies to describe the effects of HF feeding and FOS supplementation on the cardiac proteome and can therefore serve as a foundation for future work.

## Figures and Tables

**Table 1:** Composition of the experimental diets (per 1000 g).

COMPOSITION	CON	HF	HF-FOS
Ingredient (g)			
Casein (protein)	200	190	190
L-Cystine	3	3	3
Corn Starch	387.5	173.5	172.6
Maltodextrin 10	122	68.3	67.6
Sucrose	100	283.3	282.6
Cellulose, BW200	70	70	40
Fructooligosaccharide	0	0	32.2
Soybean Oil	70	120.3	120.3
Milk Fat, Anhydrous	0	44.2	44.2
tBHQ	0.014	0.014	0.014
Mineral Mix S10022G	35	35	35
Nutrient (g)			
Protein	179.0	170.2	170.2
Carbohydrate (digestible)	609.5	524.9	522.8
Fat	70.0	164.5	164.5
Fibre	70.0	70.0	72.2
Nutrient (kcal)			
Protein	716	681	681
Carbohydrate	2438	2100	2091
Fat	630	1481	1481
Total	3784	4261	4253

CON, control diet (AIN-93G); HF, high fat diet (AIN-93G + high fat content); HF-FOS, high-fat diet supplemented with fructooligosaccharides.

**Table 2:** Enriched gene ontology (GO) terms and KEGG pathways in the list of differentially expressed proteins in HF hearts compared to CON.

Biological Theme *	Gene Names ^‡^	GO and KEGG Pathways ^†^	*p*-Value ^§^
Metabolism (cholesterol)	**apolipoprotein A1 *(Apoa1)*** **apolipoprotein A4 *(Apoa4)*** apolipoprotein E *(Apoe)* **catalase *(Cat)*** **hydroxysteroid (17-beta) dehydrogenase *4 (Hsd17b4)***	Cholesterol metabolic process	3.3 × 10^-4^
		KEGG:	
		Fat digestion and absorption	3.7 × 10^-1^
		Peroxisome	1.6 × 10^-1^
		Vitamin digestion and absorption	3.6 × 10^-1^
Metabolism (lipid)	**apolipoprotein A1 *(Apoa1)*** **apolipoprotein A4 *(Apoa4)***	VLDL remodelling	3.5 × 10^-3^

	apolipoprotein E (Apoe)	Positive regulation of cholesterol esterification	3.5 × 10^-3^
		HDL particle assembly	3.5 × 10^-3^
		Peripheral nervous system axon regeneration	6.5 × 10^-3^
		Reverse cholesterol transport	6.5 × 10^-3^
		Phospholipid efflux	7.5 × 10^-3^
		Triglyceride catabolic process	2.1 × 10-^2^
		Triglyceride homeostasis	2.0 × 10^-2^
		Cholesterol Efflux	1.9 × 10^-2^
		Lipoprotein metabolic process	1.9 × 10^-2^
		KEGG:	
		Fat digestion and absorption	3.7 × 10^-1^
		Vitamin digestion and absorption	3.6 × 10^-1^

CON, control diet; HF, high fat diet. * The Functional Annotation Clustering general theme as indicated by DAVID. ^†^ GO (BP) is gene ontology (GO) biological process component (BP) and KEGG pathway is Kyoto Encyclopedia of Genes and Genomes biological pathway. ^‡^ **Gene names in bold** indicate that the protein respective to that gene was upregulated between groups; un-bolded gene names indicate downregulation of the respective protein between groups. ^§^ *p*-value of the enrichment analyses is significant at Benjamini–Hochberg *p* < 0.05.

**Table 3:** Differentially expressed proteins in heart tissue following high-fat with fructooligosaccharide vs. high-fat treatments.

Differentially Expressed Protein	Protein Accession No.	Log_2_Fold Change (HF-FOS/HF) *	*p*-Value ^†^	Gene Name
apolipoprotein A-IV (APOA4)	EDL95397.1	−0.16	<0.0001	*Apoa4*
catalase, isoform CRA_b (CAT)	EDL79667.1	−0.07	0.0011	*Cat*
creatine kinase, brain, isoform CRA_b (CKB)	EDL97457.1	−0.14	<0.0001	*Ckb*
electron transferring flavoprotein, alpha polypeptide (ETFA)	EDL95569.1	0.06	0.0010	*Etfa*
filamin, alpha (predicted), isoform CRA_b (FLNA)	EDL84990.1 (+1)	−0.12	<0.0001	*Flna*
four and a half LIM domains 1, isoform CRA_b (FHL1)	EDL75140.1	−0.13	0.0003	*Fhl1*
PREDICTED: annexin A6 isoform X1 (ANXA6)	XP_017453031.1	0.09	<0.0001	*Anxa6*
preproapolipoprotein A-I (APOA1)	CAA25224.1	−0.16	<0.0001	*Apoa1*
stress-70 protein, mitochondrial (HSPA9)	NP_001094128.2	−0.06	<0.0001	*Hspa9*
myosin regulatory light chain 2, ventricular/cardiac muscle isoform (MLY2)	NP_001030329.2	0.16	0.0001	*Myl2*
PREDICTED: guanylate-binding protein 1 (GBP1)	XP_006224340.1 (+1)	0.21	0.0002	*Gbp1*
tropomyosin 4 (TPM4)	EDL90830.1	−0.16	0.0002	*Tpm4*
glycerol-3-phosphate dehydrogenase 1-like protein (GPD11)	NP_001178814.1	−0.12	<0.0001	*Gpd1l*
heat shock protein, alpha-crystallin-related, B6/ Hsp20 (HSPB6)	EDM07749.1	−0.11	0.0026	*Hspb6*
LIM and cysteine-rich domains protein 1 (LMCD1)	NP_001008562.1	−0.13	<0.0001	*Lmcd1*
myomesin-1 (MYOM1)	NP_001178513.1	0.09	<0.0001	*Myom1*
nebulette (NEB1)	NP_001178623.1	−0.09	<0.0001	*Nebl*
nexilin, isoform CRA_c (NEXN)	EDL82495.1 (+1)	−0.09	0.0004	*Nexn*
PREDICTED: cAMP-dependent protein kinase type I-alpha regulatory subunit isoform X1 (PRKAR1a)	XP_017452536.1	−0.07	0.0015	*Prkar1a*
PREDICTED: cysteine and glycine-rich protein 3 isoform X1/ cardiac LIM protein (CSRP3)	XP_006229299.1	−0.11	0.0005	*Csrp3*
PREDICTED: heat shock protein HSP 90-alpha (LOC103692716)	XP_008763191.1	−0.07	0.0013	*LOC103692716*
PREDICTED: myomesin-2 isoform X1 (MYOM2)	XP_017455650.1	0.06	<0.0001	*Myom2*
actinin alpha 2 (ACTN2)	EDM06982.1	0.1	<0.0001	*Actn2*
ROK-alpha (Rho-associated protein expression kinase) (ROCK2)	AAB37540.1 (+1)	−0.1	0.0029	*Rock2*
sarcoplasmic/endoplasmic reticulum calcium ATPase 2 isoform a (ATP2A2)	NP_001103609.1	0.04	<0.0001	*Atp2a2*
vinculin (predicted), isoform CRA_a (VCL)	EDL86257.1	−0.05	0.0002	*Vcl*
acetyl-Coenzyme A dehydrogenase, short chain, isoform CRA_a (ACADS)	EDM13909.1	0.06	0.0003	*Acads*
aldehyde dehydrogenase (ALDH1A1)	AAA96657.1	0.25	0.0004	*Aldh1a1*
kynurenine—oxoglutarate transaminase 1, mitochondrial (KYAT1)	NP_001013182.3	−0.24	< 0.0001	*Kyat1*
PREDICTED: mimitin, mitochondrial (NDUFAF2)	XP_001073799.1	−0.18	0.0014	*Ndufaf2*
prolyl endopeptidase (PREP)	EDL99674.1 (+1)	−0.16	0.0005	*Prep*
pyridine nucleotide-disulfide oxidoreductase domain-containing protein 2 (PYRDOXD2)	NP_001004261.1 (+3)	−0.27	<0.0001	*Pyroxd2*
cytochrome c-1 (predicted), isoform CRA_c (CYC1)	EDM15989.1	0.13	<0.0001	*Cyc1*
glutathione S-transferase omega 1 (GSTO1)	ACI32122.1	−0.12	0.0036	*Gsto1*
heat shock 70kDa protein 5 (glucose-regulated protein), isoform CRA_a (HSPA5)	EDL93170.1	−0.08	<0.0001	*Hspa5*
inter alpha-trypsin inhibitor, heavy chain 4, isoform CRA_a (LTIH4)	EDL88978.1	−0.12	<0.0001	*Ltih4*
kynurenine—oxoglutarate transaminase 3 (KYAT3)	NP_001015037.1	−0.13	0.0006	*Kyat3*
leucine-rich PPR motif-containing protein, mitochondrial precursor (LRPPRC)	NP_001008519.1	−0.06	<0.0001	*Lrpprc*
methylcrotonoyl-CoA carboxylase beta chain, mitochondrial (MCCC2)	NP_001012177.1	−0.07	<0.0001	*Mccc2*
methylcrotonoyl-CoA carboxylase subunit alpha, mitochondrial (MCCC1)	NP_001009653.1	−0.09	<0.0001	*Mccc1*
muscle glycogen phosphorylase (PYGM)	EDM12601.1	0.07	<0.0001	*Pygm*
Pgm1 protein, partial (PGM1)	AAI28704.1	−0.05	0.0003	*Pgm1*
PREDICTED: isobutyryl-CoA dehydrogenase, mitochondrial isoform X2 (ACAD8)	XP_003754442.1	−0.07	0.0025	*Acad8*
PREDICTED: triosephosphate isomerase (LOC100911515)	XP_003750702.2	−0.06	<0.0001	*LOC100911515*
rCG20653 (ACOT2)	EDL81468.1	0.07	0.0002	*Acot2*
rCG45082 (C3)	EDL83571.1	−0.05	<0.0001	*C3*
Tu translation elongation factor, mitochondrial (predicted), isoform CRA_c (TUFM)	EDM17456.1	−0.05	0.0001	*Tufm*
ubiquinol cytochrome c reductase core protein 2, isoform CRA_c (UQCRC2)	EDM17618.1	0.05	0.0005	*Uqcrc2*
glutathione peroxidase 1 (GPX1)	NP_110453.3	−0.1	0.0003	*Gpx1*
arginine-tRNA-protein transferase 1 (predicted), isoform CRA_a (ATE1)	EDM17146.1	−0.21	0.0026	*Ate1*
DnaJ (Hsp40) homolog, subfamily A, member 2, isoform CRA_b (DNAJ)	EDL87492.1	−0.15	<0.0001	*DnaJ*
rCG22471, isoform CRA_b (EEF1B2)	EDL98899.1	−0.21	<0.0001	*Eef1b2*
chaperonin subunit 8 (theta) (predicted), isoform CRA_a (CCT8)	EDM10642.1	−0.06	0.0026	*Cct8*
crystallin, alpha B, isoform CRA_a (CRYAB)	EDL95479.1	−0.12	<0.0001	*Cryab*
guanosine diphosphate dissociation inhibitor 1, isoform CRA_a (GDI1)	EDL84976.1	−0.08	0.0011	*Gdi1*
heat shock protein 27 (HSPB1)	AAA41353.1	−0.12	<0.0001	*Hspb1*
peptidylprolyl isomerase F (cyclophilin F), isoform CRA_a (PPIF)	EDL75095.1	−0.07	0.0026	*Ppif*
proteasomal ATPase (rat TBP1) (PSMC3)	BAA11939.1	−0.11	0.0003	*Psmc3*
proteasome (prosome, macropain) subunit, alpha type 1, isoform CRA_d (PSMA1)	EDM17781.1	−0.09	0.0037	*Psma1*
rCG38543, isoform CRA_a (PSMA7)	EDL88834.1	−0.07	0.0013	*Psma7*
drebrin-like, isoform CRA_b (DBN1)	EDM00307.1	−0.17	0.0011	*Dbnl*
oxidative-stress responsive 1 (predicted) (OXSR1)	EDL76918.1	−0.16	0.0019	*Oxsr1*
Ehd1 protein, partial (EHD1)	AAI60908.1	−0.08	<0.0001	*Ehd1*
EH-domain containing 4 (EHD4)	EDL79933.1	−0.1	<0.0001	*Ehd4*
heat shock 27kD protein family, member 7 (cardiovascular) (HSPB7)	EDL80988.1	−0.12	0.0038	*Hspb7*
nonmuscle myosin heavy chain-A (MYH9)	AAA74950.1	−0.12	<0.0001	*Myh9*
PDZ and LIM domain 1 (elfin), isoform CRA_a (PDLIM1)	EDL94180.1	−0.1	0.0017	*Pdlim1*
PREDICTED: adenylyl cyclase-associated protein 2 isoform X1 (CAP2)	XP_006253816.1	−0.12	< 0.0001	*Cap2*
PREDICTED: beta-1-syntrophin isoform X1 (SNTB1)	XP_017450243.1	−0.11	< 0.0001	*Sntb1*
PREDICTED: spectrin alpha chain, non-erythrocytic 1 isoform X1 (SPTAN1)	XP_008759895.1	0.06	0.0004	*Sptan1*
PREDICTED: tubulin-folding cofactor B (LOC103690005)	XP_017444696.1	−0.2	0.0011	*LOC103690005*
rCG27764, isoform CRA_a (DYNC1H1)	EDL97508.1	−0.08	<0.0001	
acyl-CoA synthetase family member 2, mitochondrial precursor (ACSF2)	NP_001030123.1	0.04	0.0012	*Acsf2*
acyl-Coenzyme A dehydrogenase, very long chain, isoform CRA_c (ACADVL)	EDM04964.1	0.06	<0.0001	*Acadvl*
arginine--tRNA ligase, cytoplasmic (RARS)	NP_001099247.2	−0.11	0.0002	*Rars*
catenin beta-1 (CTNNB1)	NP_445809.2 (+1)	−0.08	0.0026	*Ctnnb1*
calsequestrin 2, isoform CRA_a (CASQ2)	EDL85514.1	−0.05	0.0037	*Casq2*
cellular nucleic acid binding protein 1, isoform CRA_b (CNBP)	EDL91294.1	−0.12	0.0036	*Cnbp*
enoyl coenzyme A hydratase 1, peroxisomal (ECH1)	EDM07870.1	−0.1	<0.0001	*Ech1*
glutamate dehydrogenase 1, isoform CRA_a (GLUD1)	EDL88881.1	−0.05	0.0019	*Glud1*
GrpE-like 1, mitochondrial (GRPEL1)	EDM00047.1	−0.1	0.0007	*Grpel1*
Hagh protein, partial (HAGH)	AAH97301.1	−0.1	0.0025	*Hagh*
Keratin 5 (KRT5)	AAH62086.1	−0.56	0.0018	*Krt5*
L-3-hydroxyacyl-Coenzyme A dehydrogenase, short chain, isoform CRA_a (HADH)	EDL82212.1	0.09	0.0002	*Hadh*
PREDICTED: glycogen phosphorylase, brain form isoform X1 (PYGB)	XP_017446988.1	0.05	0.0026	*Pygb*
PREDICTED: microtubule-associated protein 4 isoform X1 (MAP4)	XP_006243875.1	−0.1	0.001	*Map4*
PREDICTED: myosin-binding protein C, cardiac-type isoform X1 (MYBPC3)	XP_006234566.1	0.07	<0.0001	*Mybpc3*
thioredoxin 2, isoform CRA_b (TXN2)	EDM15902.1	−0.27	0.0034	*Txn2*
TPA_exp: type II keratin Kb1 (KRT1)	DAA02055.1	−0.43	0.0038	*Krt1*
transcription factor A, mitochondrial, isoform CRA_a (TFAM)	EDL97257.1	−0.1	0.0023	*Tfam*
glutamate oxaloacetate transaminase 1, isoform CRA_a (GOT1)	EDL94253.	−0.07	<0.0001	*Got1*
Gpc1 protein (GPC1)	AAH61572.1	−0.08	0.0005	*Gpc1*
isocitrate dehydrogenase 3 (NAD+) alpha, isoform CRA_a (IDH3a)	EDL95540.1	−0.06	0.0038	*Idh3a*
PREDICTED: myosin-7 isoform X2 (MYH7)	XP_006252013.1	−0.17	<0.0001	*Myh7*
ATPase family, AAA domain containing 3A (ATAD3A)	EDL81320.1	−0.17	0.0009	*Atad3a*
glyoxylase 1 (GLO1)	EDL96989.1	−0.14	0.0007	*Glo1*
PREDICTED: pre-B-cell leukemia transcription factor-interacting protein 1 isoform X4 (PBXIP1)	XP_017446382.1	−0.14	0.0012	*Pbxip1*
PREDICTED: solute carrier family 12 member 7 isoform X2 (SLC12A7)	XP_006227851.1	−0.19	<0.0001	*Slc12a7*
PREDICTED: transportin-1 isoform X1 (TNPO1)	XP_008758890.1	−0.25	0.0029	*Tnpo1*
AFG3-like protein 2 (AFG312)	NP_001128336.1	−0.08	0.0002	*Afg3l2*
annexin A5 (ANXA5)	NP_037264.1 (+ 4)	−0.12	<0.0001	*Anxa5*
clathrin heavy chain 1 (CLTC)	NP_062172.1	−0.06	<0.0001	*Cltc*
complement inhibitory factor H (CFH)	CAC67513.1	−0.09	<0.0001	*Cfh*
elongation factor 1-delta (EEFLD)	NP_001013122.1	−0.09	<0.0001	*Eef1d*
fibrinogen B beta chain (FGB)	AAA64866.1	−0.1	<0.0001	*Fgb*
inter-alpha-trypsin inhibitor heavy chain H3 precursor (ITIH3)	NP_059047.1 (+ 4)	−0.13	0.0033	*Itih3*
lumican (LUM)	EDM16832.1	0.06	0.0029	*Lum*
PREDICTED: dynamin-like 120 kDa protein, mitochondrial isoform X1 (OPA1)	XP_006248559.1	−0.07	<0.0001	*Opa1*
PREDICTED: fibrinogen alpha chain isoform X1 (FGA)	XP_006232594.1	−0.13	<0.0001	*Fga*
rat ribosomal protein L13a (RPL13A)	CAA48343.1	−0.11	0.0010	*Rpl13a*
rCG27551, isoform CRA_b (DSTN)	EDL95182.1	−0.1	0.0017	*Dstn*
rCG35863 (LAP3)	EDL99928.1	−0.11	<0.0001	*Lap3*
selenium-binding protein 1 (LOC103689947)	NP_001316822.1	−0.06	0.0023	*LOC103689947*
PREDICTED: similar to RIKEN cDNA 2310039E09 (CAVIN4)	EDL78187.1	−0.13	0.0023	*Cavin4*
Sucla2 protein, partial; succinate-coA ligase subunit beta (SUCLA2)	AAI66998.1	−0.06	0.0003	*Sucla2*
thioredoxin-like 5 (predicted), isoform CRA_b (TXNDC17)	EDM05095.1	−0.09	0.0014	*Txndc17*

HF-FOS, high-fat diet supplemented with fructooligosaccharide; HF, high fat diet. * Log_2_Fold change of each protein (HF-FOS/HF). -^ve^ values indicate downregulation and + ^ve^ values indicate upregulation of the protein in the HF-FOS group compared to HF. ^†^ *p*-value of the Mann–Whitney test with Benjamini–Hochberg multiple corrections test. Significant if *p* < 0.0040.

**Table 4:** Enriched GO terms and KEGG pathways of differentially expressed proteins between HF-FOS and HF diet groups in heart tissue according to biological theme.

Biological Theme *	Gene Names ^‡^	GO Terms and KEGG Pathways ^†^	*p*-Value ^§^
Fatty acid homeostasis	acyl-CoA dehydrogenase family, member 8 *(Acad8)* **acyl-CoA dehydrogenase, C-2 to C-3 short chain *(Acads)*** **acyl-CoA dehydrogenase, very long chain *(Acadvl)*** apolipoprotein A4 (*Apoa4*) **electron transfer flavoprotein alpha subunit *(Etfa)***	GO-lipid homeostasis	3.0 × 10^−2^
	acyl-CoA dehydrogenase family, member 8 (*Acad8*) **acyl-CoA dehydrogenase, C-2 to C-3 short chain *(Acads)*** **acyl-CoA dehydrogenase, very long chain *(Acadvl)*** **electron transfer flavoprotein alpha subunit *(Etfa)***	GO-fatty acid beta-oxidation using acyl-CoA dehydrogenase	3.8 × 10^-2^
Cardiac muscle regulation	calsequestrin 2 (*Casq2*) cysteine and glycine rich protein 3 (*Csrp3*) **myosin binding protein C, cardiac *(Mybpc3)*** myosin heavy chain 7 (*Myh7*) **myosin light chain 2 *(Myl2)***	GO-cardiac muscle contraction	3.6 × 10^-2^
	**actinin alpha 2 *(Actn2)*** **myomesin 1 *(Myom1)*** **myomesin 2 *(Myom2)*** **myosin binding protein C, cardiac *(Mybpc3)*** myosin heavy chain 7 (*Myh7*) tropomyosin 4 (*Tpm4*)	GO-muscle contraction	6.6 × 10^-3^
Carbohydrate metabolism	**acyl-CoA dehydrogenase, C-2 to C-3 short chain *(Acads)*** catalase (*Cat*) glutamate dehydrogenase 1 (*Glud1*) glutamic-oxaloacetic transaminase 1 (*Got1*) isocitrate dehydrogenase 3 (NAD+) alpha (*Idh3a*) succinate-CoA ligase ADP-forming beta subunit t (*Sucla2*) triosephosphate isomerase-like (*LOC100911515*)	KEGG-carbon metabolism	7.3 × 10^-2^
	glycerol-3-phosphate dehydrogenase 1-like (*Gpd1l*) glyoxalase 1(*Glo1*) hydroxyacyl glutathione hydrolase (*Hagh*) phosphoglucomutase 1 (*Pgm1*) **phosphorylase, glycogen, muscle *(Pygm)*** **phosphorylase, glycogen; brain *(Pygb)***	GO-carbohydrate metabolic process	8.8 × 10^-2^
Protein processes	GrpE-like 1, mitochondrial (*Grpel1*) chaperonin containing TCP1 subunit 8 (*Cct8*) crystallin, alpha B (*Cryab*) heat shock protein HSP 90-alpha (*LOC103692716*) heat shock protein family A member 9 (*Hspa9*) peptidylprolyl isomerase F (*Ppif*) thioredoxin 2 (*Txn2*)	KEGG-Protein folding	2.1 × 10^-2^
Adhesion and cytoskeletal regulation	PDZ and LIM domain 1 (*Pdlim1*) arginyl-tRNA synthetase (*Rars*) chaperonin containing TCP1 subunit 8 (*Cct8*) drebrin-like protein (*Dbnl*) eukaryotic translation elongation factor 1 delta (*Eef1d*) heat shock protein family A member 5 *(Hspa5)* **spectrin, alpha, non-erythrocytic 1 *(Sptan1)***	GO-cell-cell adhesion	4.5 × 10^-2^

HF-FOS, high-fat diet with fructooligosaccharides; HF, high-fat diet. * The functional annotation clustering general theme as indicated by Database for Annotation, Visualization and Integrated Discovery (DAVID). ^†^ GO is gene ontology biological process component and KEGG is Kyoto Encyclopedia of Gene and Genomes biological pathway. ^‡^ **Gene names in bold** indicate that the protein respective to that gene was upregulated between groups; un-bolded gene names indicate downregulation of the respective protein between groups. ^§^ *p*-value of the enrichment analyses is significant at Benjamini–Hochberg *p* < 0.05.

## Supplementary Materials

The following are available online at https://www.mdpi.com/2072-6643/12/11/3404/s1, Supplementary Tables S1 and S2.
