# Supplementary material for: Effects of High-Fat Diet Induced Obesity and Fructooligosaccharide Supplementation on Cardiac Protein Expression
Source: Nutrients. 2020 Nov 5;12(11):3404. doi: 10.3390/nu12113404 (PMC7694524; doi:10.3390/nu12113404)
Supplement: Supplementary file 1 [file nutrients-12-03404-s001.zip › Nutrients-965138 Suppl. Table2.docx]

**Supplemental Table 2.** Differentially expressed proteins in heart tissue following high-fat and control diets.

| Differentially Expressed Protein | Protein Accession No. | Log_2_Fold Change (HF/CON)* | *p-*value† | Gene Name |
| --- | --- | --- | --- | --- |
| acyl-CoA synthetase family member 2, mitochondrial precursor (ACSF2) | NP_001030123.1 | -0.11 | < 0.0001 | *Acsf2* |
| Aldh4a1 protein, partial (ALDH4A1) | AAI68153.1 (+1) | -0.09 | < 0.0001 | *Aldh4a1* |
| apolipoprotein A-IV (APOA4) | EDL95397.1 | 0.13 | < 0.0001 | *Apoa4* |
| apolipoprotein E, isoform CRA_c (APOE) | EDM08152.1 | -0.16 | < 0.0001 | *Apoe* |
| catalase, isoform CRA_b (CAT) | EDL79667.1 | 0.13 | < 0.0001 | *Cat* |
| Coq9 protein, partial (COQ9) | AAI04703.1 (+1) | -0.09 | < 0.0001 | *Coq9* |
| creatine kinase, brain, isoform CRA_b (CKB) | EDL97457.1 | 0.1 | < 0.0001 | *Ckb* |
| electron transferring flavoprotein, alpha polypeptide (ETFA) | EDL95569.1 | -0.07 | 0.0008 | *Etfa* |
| enoyl coenzyme A hydratase 1, peroxisomal (ECH1) | EDM07870.1 (+2) | 0.11 | < 0.0001 | *Ech1* |
| filamin, alpha (predicted), isoform CRA_b (FLNA) | EDL84990.1 (+1) | 0.12 | < 0.0001 | *Flna* |
| four and a half LIM domains 1, isoform CRA_b (FHL1) | EDL75140.1 | 0.15 | 0.0005 | *Fhl1* |
| hydroxysteroid (17-beta) dehydrogenase 4, isoform CRA_b, partial (HSD17B4) | EDM14436.1 (+1) | 0.1 | 0.0004 | *Hsd17b4* |
| isovaleryl coenzyme A dehydrogenase, isoform CRA_a (IVD) | EDL79883.1 | -0.15 | < 0.0001 | *Ivd* |
| methylmalonate semialdehyde dehydrogenase (ALDH6A1) | AAA41638.1 (+2) | -0.05 | < 0.0001 | *Aldh6a1* |
| PREDICTED: adenylosuccinate synthetase isozyme 1 (ADSSL1) | XP_003750285.1 | -0.07 | 0.0006 | *Adssl1* |
| PREDICTED: annexin A6 isoform X1 (ANXA6) | XP_017453031.1 | -0.08 | < 0.0001 | *Anxa6* |
| PREDICTED: atypical kinase ADCK3, mitochondrial isoform X1 (COQ8A) | XP_006250449.1 | -0.09 | 0.0002 | *Coq8a* |
| PREDICTED: NAD(P) transhydrogenase, mitochondrial isoform X1 (NNT) | XP_008758996.1 | -0.21 | < 0.0001 | *Nnt* |
| preproapolipoprotein A-I (APOA1) | CAA25224.1 | 0.16 | < 0.0001 | *Apoa1* |
| rCG20683, isoform CRA_b, also known as Maleylacetoacetate isomerase (GSTZ1) | EDL81627.1 (+1) | -0.25 | < 0.0001 | *Gstz1* |
| rCG49564, isoform CRA_a (CDH2) | EDL76078.1 | 0.12 | 0.0005 | *Cdh2* |
| PREDICTED: tripartite motif protein 50 (TRIM72) | EDM17199 | -0.13 | < 0.0001 | *Trim72* |
| stress-70 protein, mitochondrial (HSPA9) | NP_001094128.2 | 0.05 | 0.0002 | *Hspa9* |

CON, control diet; HF, high fat diet. *Log_2_Fold Change of each protein (HF/Control). -^ve^ values indicate downregulation and +^ve^ values indicate upregulation of the protein in the HF group compared to CON. † *p-*value of the Mann-Whitney test with Benjamini–Hochberg multiple corrections test. Significant if *p*<0.0008.
